# The initiation of embryo growth in imbibed celery mericarps marks a key mechanism by which temperature signals are integrated to regulate germination timing

**DOI:** 10.1093/jxb/eraf326

**Published:** 2025-07-21

**Authors:** Matthew Walker, Kazumi Nakabayashi, Frances Gawthrop, Gerhard Leubner-Metzger

**Affiliations:** Department of Biological Sciences, Royal Holloway University of London, Egham, Surrey TW20 0EX, UK; Tozer Seeds Ltd, Cobham, Surrey KT11 3EH, UK; Department of Biological Sciences, Royal Holloway University of London, Egham, Surrey TW20 0EX, UK; Department of Agro-environmental Science, Obihiro University of Agriculture and Veterinary Medicine, Obihiro, Hokkaido 080-8555, Japan; Tozer Seeds Ltd, Cobham, Surrey KT11 3EH, UK; Department of Biological Sciences, Royal Holloway University of London, Egham, Surrey TW20 0EX, UK; Laboratory of Growth Regulators, Institute of Experimental Botany, Czech Academy of Sciences and Faculty of Science, Palacký University Olomouc, Olomouc CZ-78371, Czech Republic; University of Szeged, Hungary

**Keywords:** Abundant endosperm, *Apium graveolens* (celery), chilling temperature, embryo growth, morphological dormancy, temperature–hormone interactions, thermal time modelling, thermoinhibition, underdeveloped (small) embryo

## Abstract

Relative embryo size (embryo:seed length ratio) is a key trait in which the internal morphology of mature seeds differs. It has shaped the angiosperm history at major evolutionary and climatic events, but its adaptive significance and role in dormancy are unknown. We investigated *Apium graveolens* (celery) morphologically dormant (MD) fruits, which have underdeveloped (small) embryos embedded in abundant endosperm tissue, for their mechanisms in response to non-optimal colder and warmer temperatures. To germinate, the underdeveloped embryo must first grow inside the endosperm to reach a critical relative embryo size. Distinct hormone–temperature interactions and molecular mechanisms underpinned the reduced embryo growth in response to suboptimal and supraoptimal temperatures. Thermoinhibition (29 °C) inhibited germination by surpressing the initiation of embryo growth in a gibberellin (GA)–abscisic acid (ABA)-regulated manner. This included inhibited endo-β-1,4-mannanase, expansin, and auxin biosynthesis gene expression. In contrast to this, during chilling and across the entire suboptimal temperature range (6–20 °C), the initiation of embryo growth was delayed in a thermal time-compliant manner, as was the expression of GA-induced genes important for ABA-insensitive endosperm degradation and embryo growth. The thermal–hormonal control of germination in seeds with underdeveloped embryos (MD) constitutes a unique programme distinct from seeds with fully developed embryos.

## Teaser

Non-optimal cold/warm temperatures inhibit germination of morphologically dormant seeds by distinct mechanisms; heat blocks within-seed growth of the underdeveloped (small) embryo, while chilling slows down embryo growth to reach the critical size required for germination.

## Introduction

The success of the seed plants in terrestrial ecosystems can, to a large extent, be attributed to seed dormancy as an innate seed property which evolved to time germination and subsequent seedling growth in a diversity of climates and habitats (Finch-Savage and Leubner-Metzger, 2006; Baskin and Baskin, 2014; Finch-Savage and Footitt, 2017). Seed dormancy is an adaptive trait with a diversity of mechanisms providing blocks to the completion of germination under environmental conditions that might otherwise be considered favourable for its occurrence if a seed were non-dormant. Global biogeographic patterns reveal that climate shapes the germination niche of temperate plants, with temperature seasonality as the main driving factor (Fernandez-Pascual *et al.*, 2019; Carta *et al.*, 2022). Seed dormancy was the dominating state during the evolutionary history of seed plants, and dormancy transitions had a significant relationship with paleotemperatures (Zhang *et al.*, 2022; Rosbakh *et al.*, 2023). Double fertilization evolved as a hallmark of angiosperm reproduction and led to the formation of endosperm tissue in addition to the embryo. The endosperm is, depending on the species, retained or partially/fully obliterated by incorporating the nutrients into the storage cotyledons (Forbis *et al.*, 2002; Finch-Savage and Leubner-Metzger, 2006; Linkies *et al.*, 2010). The internal morphology of mature angiosperm seeds therefore differs in their relative embryo sizes (ES; ‘embryo to seed’ size ratio) which represents a key trait in the seed morphospace (Forbis *et al.*, 2002; Baskin and Baskin, 2014; Carta *et al.*, 2024). Recent work by Vandelook and Carta (2025) identified that major shifts in the ES ratios occurred early in the evolutionary history of angiosperms, but very little is known about the adaptive significance of relative embryo size, its ecological role, and its contribution to seed dormancy mechanisms.

Underdeveloped (small) embryos embedded in abundant endosperm tissue of mature seeds, termed morphological (MD) or morphophysiological (MPD) dormancy, were proposed to be the ancestral state in seed dormancy evolution (Willis *et al.*, 2014). These underdeveloped embryos must first grow inside the imbibed MD/MPD seeds to reach a critical ES ratio before germination can be completed by radicle emergence (Jacobsen *et al.*, 1976; Jacobsen and Pressman, 1979; Homrichhausen *et al.*, 2003; Walck and Hidayati, 2004; Scholten *et al.*, 2009; Vandelook *et al.*, 2012; Baskin and Baskin, 2014; Porceddu *et al.*, 2017; Zhang *et al.*, 2019; Walker *et al.*, 2021; Visscher *et al.*, 2022; Blandino *et al.*, 2025). This embryo growth within the imbibed seed from initial ES ratios of typically ≤0.3 to critical ES values of typically ∼0.8 occurs at the expense of the endosperm, which is dissolved and absorbed by the embryo to fuel its growth. While the ∼12% of extant species with MD/MPD are widespread across the phylogenetic tree, they are clearly dominant among basal angiosperm clades and rare within the Rosids (Forbis *et al.*, 2002; Baskin *et al.*, 2006; Finch-Savage and Leubner-Metzger, 2006; Willis *et al.*, 2014). Consistent with the hypothesis that small embryos and dormancy are the ancestral state of angiosperm seeds, synchrotron radiation X-ray tomographic microscopy (SRXTM) of fossil seeds from the Early Cretaceous [130–100 million years ago (MYA)] revealed that these extinct angiosperms had small embryos embedded within abundant storage tissue (Friis *et al.*, 2015), and the most basal extant angiosperms produce MD/MPD seeds (Willis *et al.*, 2014; Fogliani *et al.*, 2017; Vandelook and Carta, 2025). The ancestral ES ratios during angiosperm evolution were mostly stable at 0.1–0.2 until 145 MYA (Vandelook and Carta, 2025). Major positive shifts towards larger relative embryo sizes occurred during the Early Cretaceous (145–100 MYA) associated with the rapid radiation and diversification of the angiosperms (‘Darwin’s abominable mystery’). Afterwards, the ES ratios have been relatively high (0.5–0.9) and constant within most asterid and rosid clades, with notable exceptions towards smaller relative embryo sizes including in the Apiaceae.

The Apiaceae (carrot family) had their crown age in the Late Cretaceous, and their MD/MPD seeds represent the ancestral dormant seed state with underdeveloped (small) embryos embedded in abundant endosperm tissue (Vandelook *et al.*, 2012; Walker *et al.*, 2021; Vandelook and Carta, 2025). Physiologically dormant (PD; *Arabidopsis thaliana*) and non-dormant (ND; *Lepidium sativum*) Brassicaceae seed model systems with large relative embryo size (ES ratios ∼0.9) have been extensively studied for their dormancy and germination mechanisms (e.g. Graeber *et al.*, 2014; Yan *et al.*, 2014; Chahtane *et al.*, 2017; Finch-Savage and Footitt, 2017; Steinbrecher and Leubner-Metzger, 2017). The Apiaceae provide model systems for investigating mechanisms of the ancestral morphological dormancy classes and germination of seeds with underdeveloped embryos (Homrichhausen *et al.*, 2003; Walker *et al.*, 2021; Visscher *et al.*, 2022). In the dry single-seeded indehiscent fruit halves (mericarps, hereafter called fruits) of the Apiaceae, the endosperm is encased by the dead seed coat and pericarp (fruit coat). In earlier work (Walker *et al.*, 2021 ) with MD *Apium graveolens* (celery) fruits, we demonstrated that embryo growth occurred inside imbibed celery fruits in association with endosperm degradation until a critical embryo size (EF, ‘embryo to fruit’ size ratio) required for radicle emergence. The regulation of these processes depends on a complex interaction between gibberellin (GA), auxin, and abscisic acid (ABA) metabolism and changes in the tissue-specific sensitivities to these hormones. The embryo growth inside the fruit is not simply the completion of embryogenesis or *A. thaliana*-equivalent post-embryogenesis growth, but a distinct process as revealed by the hormonal mechanisms, embryo–endosperm interactions, and the spatiotemporal expression patterns of corresponding genes at optimal temperature. Walker *et al*. (2021) demonstrated that celery fruits provide an excellent system to study this unique programme in which complex hormonal interactions control embryo growth, endosperm degradation, and germination. We show here how suboptimal (colder) and supraoptimal (warmer) ambient temperatures generate distinct hormone–temperature interactions and alter the expression of key genes involved in the control of embryo growth and germination of MD seeds.

## Materials and methods

### Plant material

‘Fruits’ of the *Apium graveolens* L. (celery) F_1_ hybrid cultivars Victoria, Monterey, and Loretta used were harvested in 2014, kept in a company warehouse storage at 14 °C with a relative humidity of 25% sealed in foil bags, and provided for the current research work in January 2017 by Tozer Seeds Ltd (Cobham, Surrey, UK). The fruits of the Apiaceae are dry schizocarps which break into two dispersal units which are single-seeded mericarps. In the current work, we use the term ‘fruit’ to refer to the mericarp. Fruit aliquots for experimental use were stored at 4 °C in airtight containers containing silica.

### Germination assays

Germination assays were performed in Panasonic MLR-352 Environmental Test Chambers (Panasonic, Bracknell, UK) set to 6, 9, 12, 20, 22, 24, and 29 °C as indicated and continuous white light (∼100 μmol s^−1^ m^−2^). Triplicates of 50 fruits were used per treatment, with each triplicate sown into a 6 cm diameter Petri dish with two filter papers (MN713, Macherey-Nagel, Dueren, Germany) and 2 ml of autoclaved deionized water (dH_2_O). Germination was defined as visible emergence of the radicle through all the encasing tissues (pericarp and endosperm). Germination assays were performed using either dH_2_O (control), 100 µM gibberellin A_4+7_ (GA; Duchefa Biochemie, Haarlem, the Netherlands), 100 µM *cis,trans*-S(+)-ABA (Duchefa), or 10 µM fluridone (FLU; Duchefa). These hormones and inhibitors were added to the germination assays from concentrated stock solutions with either dH_2_O or, for GA_4+7_, DMSO as solvent. The germination curves of water and DMSO controls did not differ (Walker *et al.*, 2021). All treatments contained 0.1% v/v plant preservative mixture (PPM; Plant Cell Technology, Washington, USA). For the germination experiments in darkness, the Petri dishes, after adding the fruits and dH_2_O, were immediately wrapped with aluminium foil and placed into a black plastic box for incubation; complete darkness was therefore maintained throughout the experiment (Petri dishes were discarded after scoring). Germination data were compared through comparison of: (i) final (maximal) germination percentage G_max_; (ii) germination uniformity U_75–25%_; and (iii) germination rate GR_g%_ (speed). Germination uniformity was defined as the time required for the middle 50% of germination to occur (t75%–t25%), whilst the germination rate was defined as the inverse of the time required for reaching g% (with g being any number between 0 and 100) germination (1/tg%).

### Embryo growth assays and imaging

Embryo growth was quantified across the same temperature transect using fruits imbibed with either dH_2_O only (control), or with 100 µM GA or 10 µM FLU at 20 °C and 29 °C. Internal embryo growth was assessed over a time course of up to 30 d, depending on the incubation temperature, using the same incubation conditions as the germination assays. At least 50 fruits were used per time point, to measure sizes they were longitudinally cut and imaged using a Leica DCF480 digital camera attached to a Leica Mz 12,5 stereomicroscope (Leica, Wetzlar, Germany). The embryo, seed, and fruit lengths as well as the embryo widths were measured via the image analysis software ImageJ v1.6.0 (National Institute of Health, Bethesda, MA, USA). Embryo (E) sizes were represented as a ratio of the fruit (F) length (E:F ratio) to account for the embryo–fruit size association. Germinated fruits were removed, and their values replaced by a mean critical E:F ratio for radicle protrusion. The critical E:F ratio was calculated by measuring the internal embryo and fruit lengths of >50 fruits where the radicle had just protruded through the pericarp (less than a quarter of the length of the fruit). Data were assessed through calculation of the embryo growth rate (EGR), which is the inverse of the time required to reach a defined E:S ratio: (i) the initiation of embryo growth, defined as the inverse of the time to an E:F ratio of 0.35 (1/*t*_0.35_ or EGR_0.35_); (ii) the speed of embryo growth, defined as the inverse of the time to an E:F ratio of 0.55 (1/*t*_0.55_ or EGR_0.55_) which represents ∼50% of pre-germinative growth; and (iii) uniformity of embryo growth, defined as the time for the 0.35–0.75 range of E:F ratios to occur (U_0.35–0.75 E:F ratio_).

### Quantitative real-time reverse transcription–PCR

Sampling was performed using the aforementioned germination conditions (dH_2_O) with triplicates at 6, 20, and 29 °C. Whole fruits were sampled in the dry state (∼40 mg) and at 0.25, 1, 3, and 5 d of imbibition (physical time points). In addition, at 6 °C fruits were sampled at 8, 16, and 24 d (physiological time points providing the same E:S ratios as 1, 3, and 5 d at 20 °C). Quantitative real-time reverse transcription–PCR (RT–qPCR) was as in Graeber *et al*. (2011) with modifications in RNA extraction, identified target and reference genes, and primer sequences used as described in detail in Walker *et al*. (2021).

### Population-based thermal time threshold modelling and statistical analysis

The cardinal temperatures permissible for germination including base temperature (T_b_), optimal temperature (T_o_), and ceiling (maximal) temperature (T_c_), and the thermal time constants Θ were identified by population-based threshold modelling for thermal time (Finch-Savage and Leubner-Metzger, 2006; Batlla and Benech-Arnold, 2015; Bradford and Bello, 2022) as described in detail by Loades *et al*. (2023). In brief, germination rates GR_g%_— the inverse of time to germination for a given percentage of the population (1/*t*_g%_)—were plotted against temperature. Linear regression analysis was used to calculate the best goodness of fit (*R*^2^) lines in the suboptimal (colder) and supraoptimal (warmer) temperature regions. T_b_ and T_c_ were estimated from their intercepts with the *x*-axis. The estimated T_b_ fitting all fractions was used to recalculate regression lines forced through this T_b_ value for the linear lines in the suboptimal temperature range. The estimated T_c(50%)_ and equal slopes were used for the linear lines in the supraoptimal temperature range. Linear lines in the suboptimal and supraoptimal temperature ranges were used to estimate T_o_ from their intersections. The thermal time constants to germination [Θ_cold(g)_ and Θ_warm(g)_] were derived from their slopes. At suboptimal temperatures, Θ_cold(g)_ varies among individual percentage fractions and is normally distributed in the population around Θ_cold(50%)_. At supraoptimal temperatures, T_c(g)_ is normal distributed around T_c(50%)_. For the embryo growth data, E:F ratios were arcsine square root transformed for statistical testing. Non-linear regressions were fitted to the raw E:F ratio datasets, and the hillslope coefficients compared via one-way ANOVA. For all other statistical tests, data were compared using *t*-tests or non-parametric Mann–Whitney test, where appropriate. All statistical analyses were performed in GraphPad Prism v10 (GraphPad Software Inc., San Diego, CA, USA).

## Results

### Embryo growth inside morphologically dormant celery fruits is blocked by darkness and by high temperatures

**Fig. 1.**
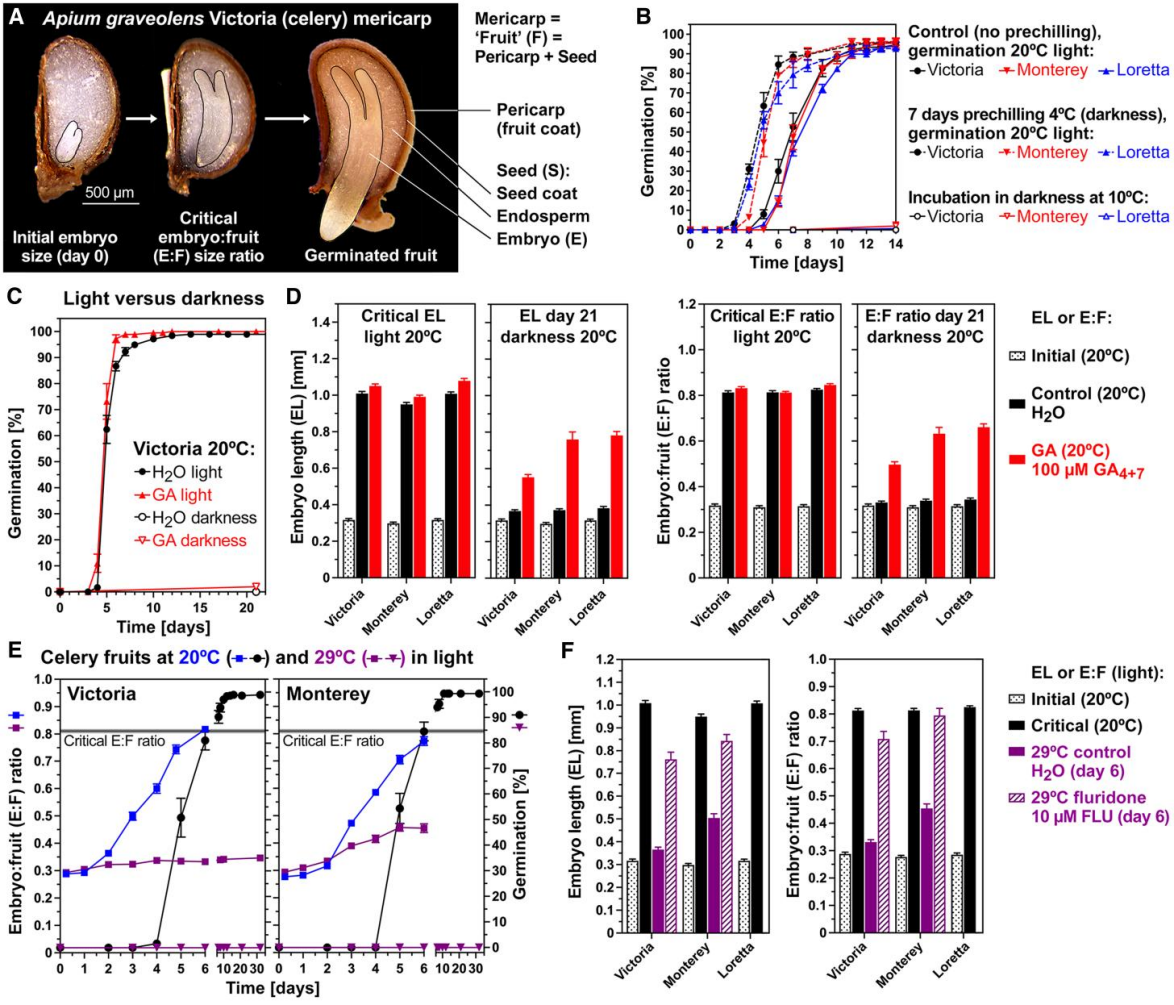
The effect of different temperatures and light/darkness on celery embryo growth and germination. (A) Microscopic images of *Apium graveolens* cultivar Victoria mericarps (hereafter termed fruits) showing that the morphological dormancy (MD) requires that the small embryo grows within the fruit. This occurs from the start of imbibition at 20 °C in continuous white light onwards to its critical size required for the completion of germination by radicle emergence; modified from Walker *et al*. (2021). (B) The effect of pre-chilling on the germination of the celery cultivars Victoria, Monterey, and Loretta. (C) The effect of light, darkness, and gibberellin (GA, 100 µM GA_4+7_) on germination and embryo growth of the three celery cultivars. (D) The effect of light, darkness, and GA on embryo length (EL) and embryo:fruit (E:F) ratios used to present the critical sizes associated with the full germination in the light, and of EL and E:F ratios in non-germinated fruits after 3 weeks incubation in darkness. (E) The effect of supraoptimal (29 °C) compared with optimal (20 °C) temperature on embryo growth and germination in the light. (F) The effect of temperature and 10 µM fluridone (FLU) to alleviate thermoinhibition on embryo growth in the light. Mean ±SEM values are presented of >50 embryos and fruits (EL and E:F ratios) or three biological replicates each consisting of 50 fruits (germination).

The dispersal units of Apiaceae (carrot family) species are dry single-seeded indehiscent fruit halves (mericarps, hereafter called fruits) with a dead outer pericarp (fruit coat) and seed coat (Fig. 1A). The *A. graveolens* (celery) fruit has MD with a small (underdeveloped) embryo embedded in abundant living endosperm tissue of the seed. In earlier work (Walker *et al.*, 2021), we demonstrated for the celery cultivar Victoria that the small embryo must first grow inside the imbibed fruit from an initial E:F length ratio of 0.32±0.01 to the critical E:F ratio of 0.81±0.01 as a requirement to complete germination by radicle emergence. Germination of the celery cultivars Victoria, Monterey, and Loretta required light and did not occur appreciably in darkness (Fig. 1). Pre-chilling treatment (4 °C) in darkness promoted subsequent germination in the light at 20 °C (Fig. 1B). This suggests that non-cold-stratified MD fruits may have retained a low level of residual physiological dormancy which does not affect G_max_, but reduces the germination speed. Germination in the light at 20 °C was associated with embryo growth inside the imbibed fruit to very similar critical embryo lengths and E:F ratios for all three cultivars (Fig. 1C, D; Table 1). The critical embryo lengths and critical E:F ratios of control (dH_2_O) and GA (100 µM GA_4+7_) treatment were very similar in the light. Imbibition of celery fruits in darkness at 20 °C inhibited germination by blocking embryo growth. Treatment with GA was not sufficient to trigger full germination in darkness within 3 weeks; the maximum germination percentages (G_max_) were 2% for Victoria (Fig. 1C), 16% for Loretto, and 50% for Monterey. In the non-germinating fruits imbibed in darkness, the GA treatment had not triggered sufficient embryo growth to reach the required critical E:F ratio (Fig. 1D). Darkness therefore inhibits celery germination by a block to embryo growth, and GA treatment cannot fully replace the light requirement.

**Table 1.** Estimated cardinal temperatures (T_o_, optimal; T_b_, base; T_c_, ceiling) and thermal time constants Θ of *Apium graveolens* cultivar Victoria mericarp germination and embryo growth imbibed across a temperature transect (6 °C–29 °C)

Process	Treatment	T_o_ (°C)	T_b_ (°C)	Θ_cold50%_ ±SD (°C·d)	T_c_ ±SD (°C)	Θ_warm50%_ (°C·d)
Embryo growth	dH_2_O (control)	21.5	2.9	61.2±16.3	29.6±2.6	−28.3
Germination	dH_2_O (control)	21.3	3.0	86.8±10.1	27.0±0.8	−40.5
Germination	100 µM GA_4+7_	22.0	3.0	79.1±9.1	29.4±0.9	−30.8
Germination	10 µM fluridone	22.0	3.0	79.6±9.6	35.3±1.6	−57.4
Germination	100 µM ABA	18.0	3.0	89.5±10.0	28.6±2.1	−66.9

**Fig. 2.**
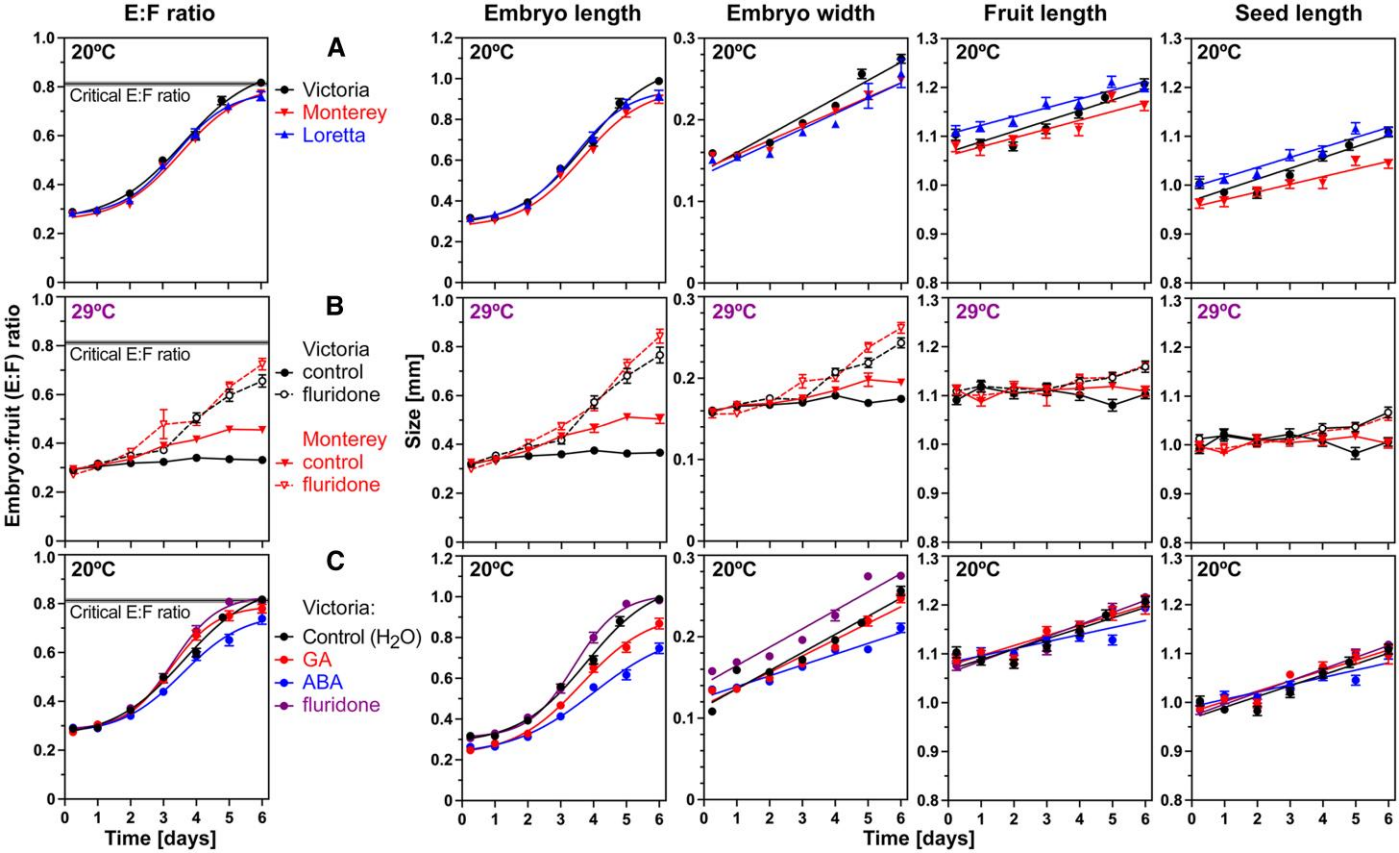
The effect of temperature and hormones on embryo growth (length and width), fruit and seed sizes, and the embryo:fruit (E:F) ratios of celery cultivars imbibed in continuous white light. (A) Celery cultivar comparison for fruit imbibition at 20 °C. (B) The effect of 10 µM fluridone (FLU) on the E:F ratios and embryo, fruit, and seed sizes of Victoria and Monterey fruits imbibed at 29 °C. (C) The effects of gibberellin (GA; 100 µM GA_4+7_), abscisic acid (100 µM ABA), and 10 µM FLU on the E:F ratios and embryo, fruit, and seed sizes of Victoria fruits imbibed at 29 °C. Mean ±SEM values are presented of >50 embryos and fruits; for (A) and (C), linear or non-linear (sigmoidal, four parameter, X is log, least square method with *R*^2^>0.8) fitted curves are presented.

In contrast to the optimal temperature (20 °C), imbibition of celery fruits in the light at 29 °C inhibited germination and embryo growth (Fig. 1E). Addition of the carotenoid/ABA biosynthesis inhibitor FLU (Yoshioka *et al.*, 1998) reversed the inhibition of germination and embryo growth at high temperatures (Fig. 1F). Embryo growth to achieve the critical embryo length and E:F ratio was restored at 29 °C by 10 µM FLU. This embryo growth inhibition by supraoptimal (warm) temperature was however not simply an ABA effect: treatment of imbibed fruits at 20 °C with physiological concentrations of ABA (up to 100 µM) did not appreciably inhibit embryo growth, but it delayed germination in a dose-dependent manner (Walker *et al.*, 2021). It is shown in Fig 2A that not only embryo length, but also embryo width, and fruit and seed length increased during fruit imbibition of all three celery cultivars at 20 °C. Imbibition at 29 °C caused thermoinhibition of celery germination, and these fruit, seed, and embryo parameters remained roughly constant (Fig. 2B). Treatment with FLU released the thermoinhibition, and this was associated with embryo growth in length and width. Finally, although ABA delayed celery germination, neither ABA, GA, nor FLU appreciably affected embryo, fruit, and seed growth of celery fruits imbibed at 20 °C (Fig. 2C). Supraoptimal (warm) temperature therefore inhibits celery germination by a block to embryo growth. This thermoinhibition is released by FLU, but ABA does not inhibit embryo growth at optimal temperatures.

### Chilling delays embryo growth, and distinct hormone–temperature interactions control germination

**Fig. 3.**
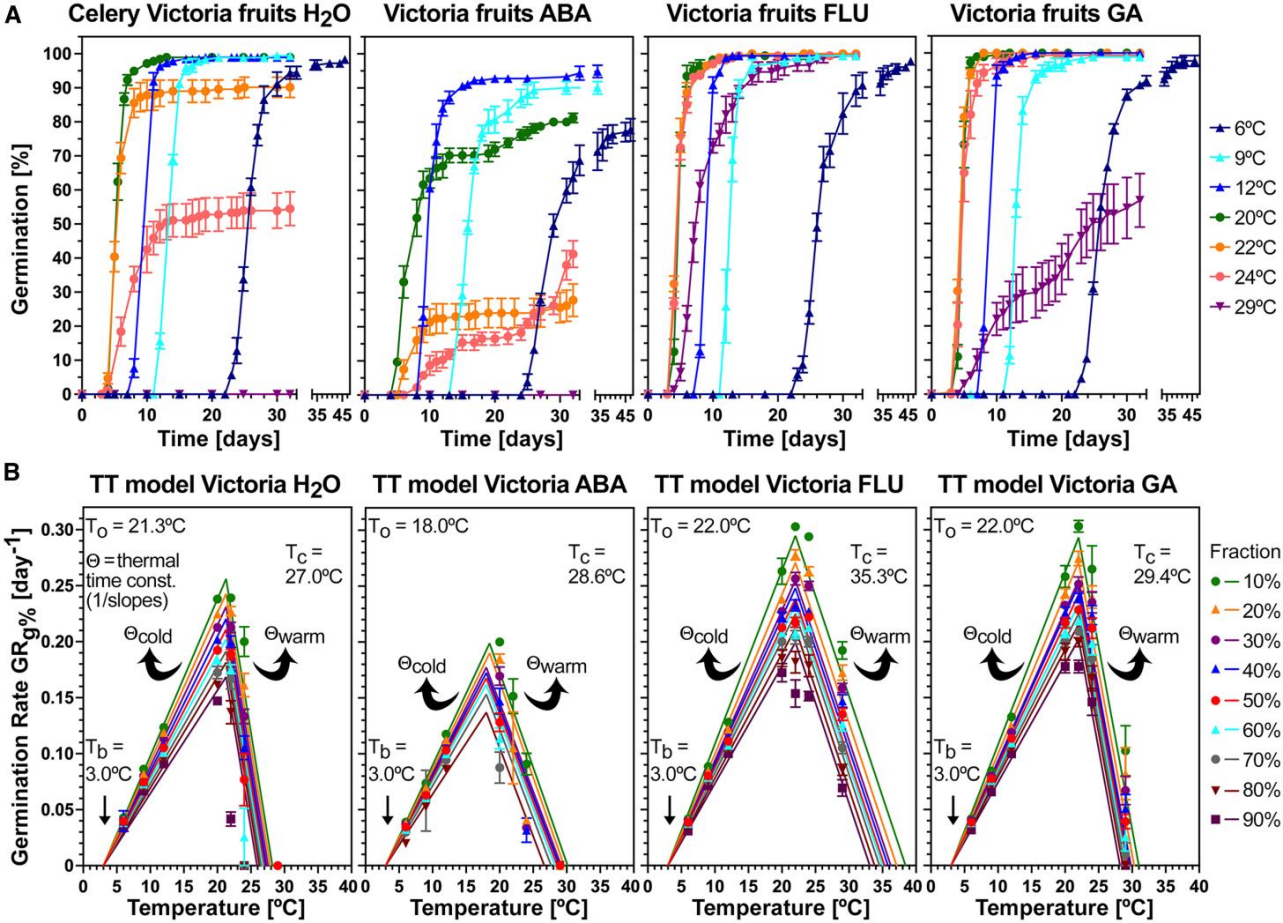
The effect of temperature–hormone interactions on the germination of celery cultivar Victoria fruits imbibed in continuous white light. (A) Germination scored as radicle emergence over a temperature transect from 6 °C to 29 °C of fruits imbibed in (from left to right) dH_2_O (control), abscisic acid (100 µM ABA), fluridone (10 µM FLU), and gibberellin (GA; 100 µM GA_4+7_). (B) Population-based thermal time (TT) models of the germination temperature responses in (A). Germination rates (GR_g%_) at the different temperatures and treatments were calculated for the fractions indicated, and repeated linear regression analysis was used to estimate the cardinal temperatures T_b_ (base), T_o_ (optimal), and T_c_ (ceiling or maximal) in °C, and these and the TT constants Θ_cold_ and Θ_warm_ in °C·d (derived fom the slopes of the regression lines; see the Materials and methods for details) are indicated and listed in Table 1. Mean ±SEM values are presented of three biological replicates each consisting of 50 fruits.

**Fig. 4.**
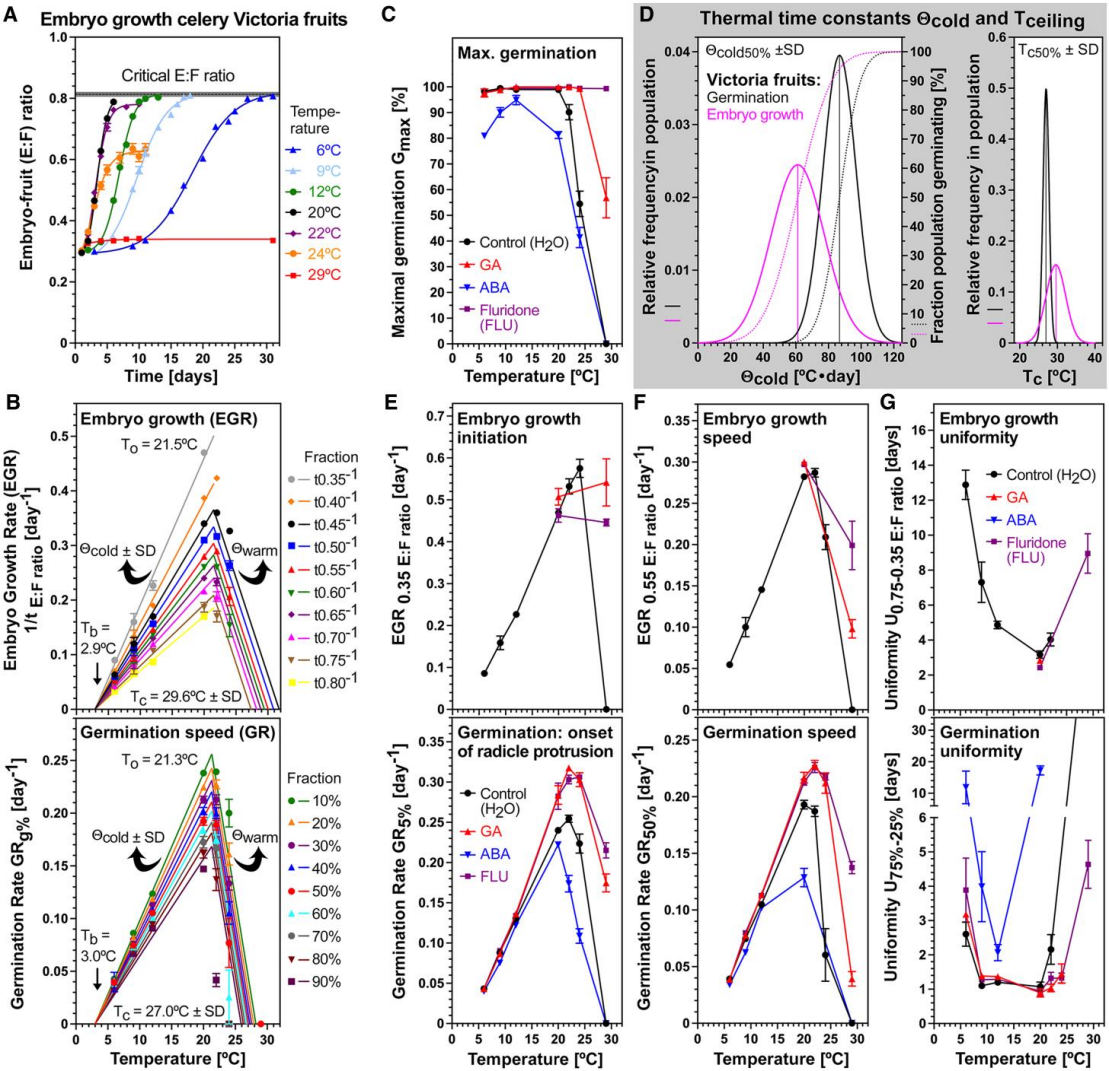
The effect of temperature–hormone interactions on the embryo growth of celery cultivar Victoria fruits imbibed in continuous white light. (A) The effect of temperature on the embryo growth in dH_2_O presented as the embryo:fruit (E:F) ratios. The critical E:F ratio required for the completion of germination is indicated. (B) Population-based thermal time (TT) model of the embryo growth [top; derived from data in (A)] and germination (bottom; derived from data in Fig. 3A) temperature responses in dH_2_O. Embryo growth rates (EGR_E:S ratio_) and germination rates (GR_g%_) were derived from the times to reach a defined fraction E:S ratio or germination percentage, respectively. The estimated cardinal temperatures T_b_ (base), T_o_ (optimal), and T_c_ (ceiling or maximal) in °C, and the TT constants Θ_cold_ and Θ_warm_ in °C·d (derived fom the slopes of the regression lines; see the Material and methods for details) are indicated and listed in Table 1. (C) The effect of temperature on the maximal germination percentages (G_max_) of Victoria fruits imbibed in dH_2_O (control), abscisic acid (100 µM ABA), fluridone (10 µM FLU), and gibberellin (GA; 100 µM GA_4+7_). (D) Frequency distribution of the TT constants Θ_cold50%_ ±SD and maximal temperatures T_c50%_ ±SD for embryo growth and germination as derived from the TT models in (B). (E–G) The effect of temperature–hormone interactions on the initiation (E), speed (F), and uniformity (G) of embryo growth (top panels) and germination (bottom panels) of Victoria fruits imbibed in the light across the temperature transect. Mean ±SEM values are presented of >50 embryos and fruits (EL and E:F ratios) or three biological replicates each consisting of 50 fruits (germination).

To examine how temperature and hormones interact to affect celery germination, we performed germination assays with the cultivar Victoria across the entire temperature transect (Fig. 3). High maximal germination percentages (G_max_ >98%) were recorded across the suboptimal (6–20 °C) temperature range (Fig. 3A). Above the temperature optimum range (20–22 °C), G_max_ rapidly declined, with ∼55% at 24 °C and 0% observed by 29 °C. Population-based thermal time modelling revealed that the estimated cardinal temperatures for the germination of the Victoria cultivar were T_b_ = 3.0 °C (base temperature), T_o_ = 21.3 °C (optimal temperature), and T_c_=27.0 °C (ceiling or maximal temperature), and the thermal time constant for the suboptimal temperature range Θ_cold50%_ ±SD was 86.8±10.1 °C·d (Fig. 3B; Table 1). To assess possible roles for GA and ABA in regulating germination responses to different temperatures, we imbibed Victoria fruits with GA, ABA, or FLU (Figs 3, 4). Over the entire 6–20 °C temperature range, there were no appreciable effects of either GA or FLU on G_max_ and T_b_. Above 20 °C, all germination traits were promoted by GA and FLU, with G_max_ at 29 °C increasing from 0% with dH_2_O to ∼100% and ∼60% with FLU and GA, respectively (Figs 3A, 4C). GA and FLU increased the germination speed (GR_g%_) in the optimal and supraoptimal temperature range, but did not appreciably shift T_o_ of the dH_2_O control (21.3 °C) (Figs 3B, 4E). In contrast to this, exogenous ABA lowered T_o_ to 18.0 °C and decreased the GR_g%_ at optimal and supraoptimal temperatures, but it did not appreciably affect GR_g%_ in the suboptimal temperature range and did not change T_b_ (Fig. 3B; Table 1). The effects of ABA and GA on T_c_ of the dH_2_O control (27.0 °C) were small, but FLU increased T_c_ to 35.3 °C (Fig 3B, 4E). Taken together (Figs 3, 4), the roles of ABA and GA in the celery germination process differ mechanistically between temperatures conferring chilling (6–12 °C), optimal (20–22 °C), and thermoinhibition (29 °C) responses.

To establish how temperature regulates embryo growth inside imbibed MD celery fruits, we quantified it across the temperature transect, expressing embryo growth as a ratio of embryo length and fruit length (E:F ratio) (Fig. 4A). No significant increase in E:F ratio was observed at 29 °C, with ratios between ∼0.32 and ∼0.34 recorded across the entire 31 d time window (*P*>0.05). The initiation of embryo growth occurred earliest (at ∼1.8 d) at 22–24 °C; below this temperature, initiation was gradually delayed, and was taking ∼10 d longer at 6 °C (Fig. 4A). The speed of embryo growth to reach an E:F ratio of 0.55, namely ∼50% of the growth of the embryo, was fastest at 22–24 °C where it was achieved by ∼3.5 d. Below this temperature, the speed of embryo growth gradually declined, taking ∼18 d at 6 °C (Fig. 4A). As for celery fruit germination (Fig. 3), a thermal time modelling approach was used to comparatively study how embryo growth responds to the entire temperature transect (Fig. 4B). This approach involved calculating embryo growth rates (EGR_E:S ratio_) which are, like GR_g%_ for germination, the inverse of the times to reach a defined E:S ratio. Embryo growth initiation and speed were represented by EGR_0.35 E:S ratio_ and EGR_0.55 E:S ratio_ (i.e. the inverse of the times required to reach E:S ratios of 0.35 and 0.55, respectively) (Fig. 4B, E, F). Subsequently segmental linear regressions were fitted, allowing estimation of the cardinal temperatures (T_b_, T_o_, and T_c_) for embryo growth. The segmental linear regressions fitted well, with the embryo growth model regressions, exhibiting *R*^2^ values of >0.97 for EGR_0.35 E:S ratio_ to EGR_0.80 E:S ratio_ in the suboptimal and optimal 6–22 °C range, and for the EGR_0.55 E:S ratio_ in the supraoptimal temperature range, while *R*^2^ values of >0.88 were obtained for the median GR_50%_ regression lines in the germination model (Fig. 4B). Thermal time modelling revealed that the estimated cardinal temperatures for embryo growth of the Victoria cultivar were T_b_ = 2.9 °C, T_o_ = 21.5 °C, and T_c_ = 28.8 °C (Fig. 4B; Table 1). The base and optimal temperatures for embryo growth and germination were therefore found to be identical in the Victoria MD fruit model. The thermal time constant for embryo growth in the suboptimal temperature range Θ_EGR0.55 E:S ratio_ ±SD was 61.2±16.3 °C·d and differed from Θ_cold50%_ ±SD (86.8±10.1 °C·d) in the germination model in that it was shifted to lower values with a larger SD (Fig. 4D). Compared with germination, embryo growth therefore responds ∼1.4-fold faster to an increase in temperature in the suboptimal range. In the supraoptimal range, T_c50%_ of embryo growth was ∼2.6 °C higher compared with germination (Fig. 4D).

**Fig. 5.**
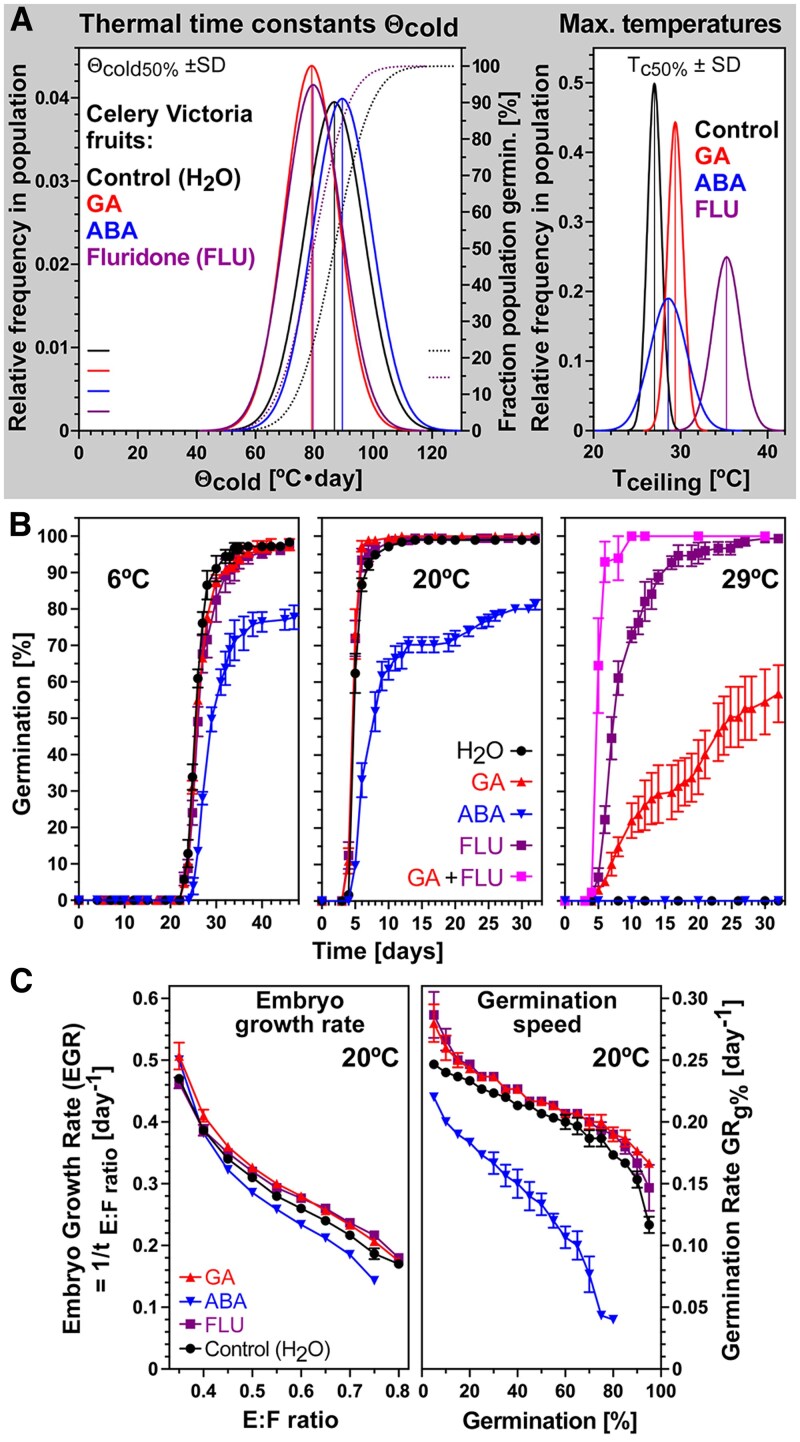
The distinct effects of hormones at suboptimal (6 °C), optimal (20 °C), and supraoptimal (29 °C) temperatures of celery cultivar Victoria fruits imbibed in continuous white light. (A) The effects of abscisic acid (100 µM ABA), fluridone (10 µM FLU), and gibberellin (GA; 100 µM GA_4+7_) on the frequency distribution of the TT constants Θ_cold50%_ ±SD and maximal temperatures T_c50%_ ±SD for germination as derived from the thermal time models in Fig. 3B. (B) The effects of GA, ABA, and FLU on germination at suboptimal (6 °C), optimal (20 °C), and supraoptimal (29 °C) temperatures. (C) The distinct effects of GA, ABA, and FLU on the embryo growth rates (EGR_xE:S ratio_; left panel) and germination rates (GR_x%_; right panel) on Victoria fruits imbibed at 20 °C. Mean ±SEM values are presented of three biological replicates each consisting of 50 fruits.

Germination traits at suboptimal temperatures were not appreciably affected by GA and FLU treatment, but they were at optimal and supraoptimal temperatures (Figs 3, 4E–G). We therefore compared the effects of these treatments on embryo growth at optimal (20 °C) and supraoptimal (29 °C) temperatures. Embryo growth initiation (EGR_0.35 E:S ratio_) and speed (EGR_0.55 E:S ratio_) were not affected by GA and FLU at 20 °C, but they were drastically enhanced at 29 °C (Fig. 4E, F). Germination onset (GR_5%_) and speed (GR_50%_) were enhanced at both temperatures. Embryo growth uniformity was best at 20 °C and was ∼9.7 d less uniform at 6 °C, and also less uniform in FLU-treated fruits at 29 °C (Fig. 4G). Compared with embryo growth, germination uniformity was best over the entire 9–20 °C range, and GA and FLU improved germination uniformity at supraoptimal temperatures. Treatment of imbibed Victoria fruits with GA and FLU did not appreciably affect germination in the suboptimal temperature range (Figs 3, 4), and Θ_cold50%_ was slightly lowered (Fig. 5A). In contrast to this, they affected germination at supraoptimal temperatures by increasing T_c_ (27.0 °C for H_2_O) which was an increase by 5.3 °C to 35.3 °C in the case of FLU (Fig. 5A). At optimal temperature (20 °C) ABA had a major effect on reducing germination speed across all GR fractions, while it did not appreciably affect embryo growth across all EGR fractions (Fig. 5B). Because of their distinct effects of GA, FLU, and ABA on the chilling (6 °C) and thermoinhibition (29 °C), germination (Fig. 5B), and embryo growth (Fig. 4), these temperatures were chosen to comparatively analyse the temperature responses on the expression of key genes identified during celery germination at optimal (20 °C) temperature (Walker *et al.*, 2021).

### Distinct temperature-controlled expression patterns of hormone- and cell-wall related genes

**Fig. 6.**
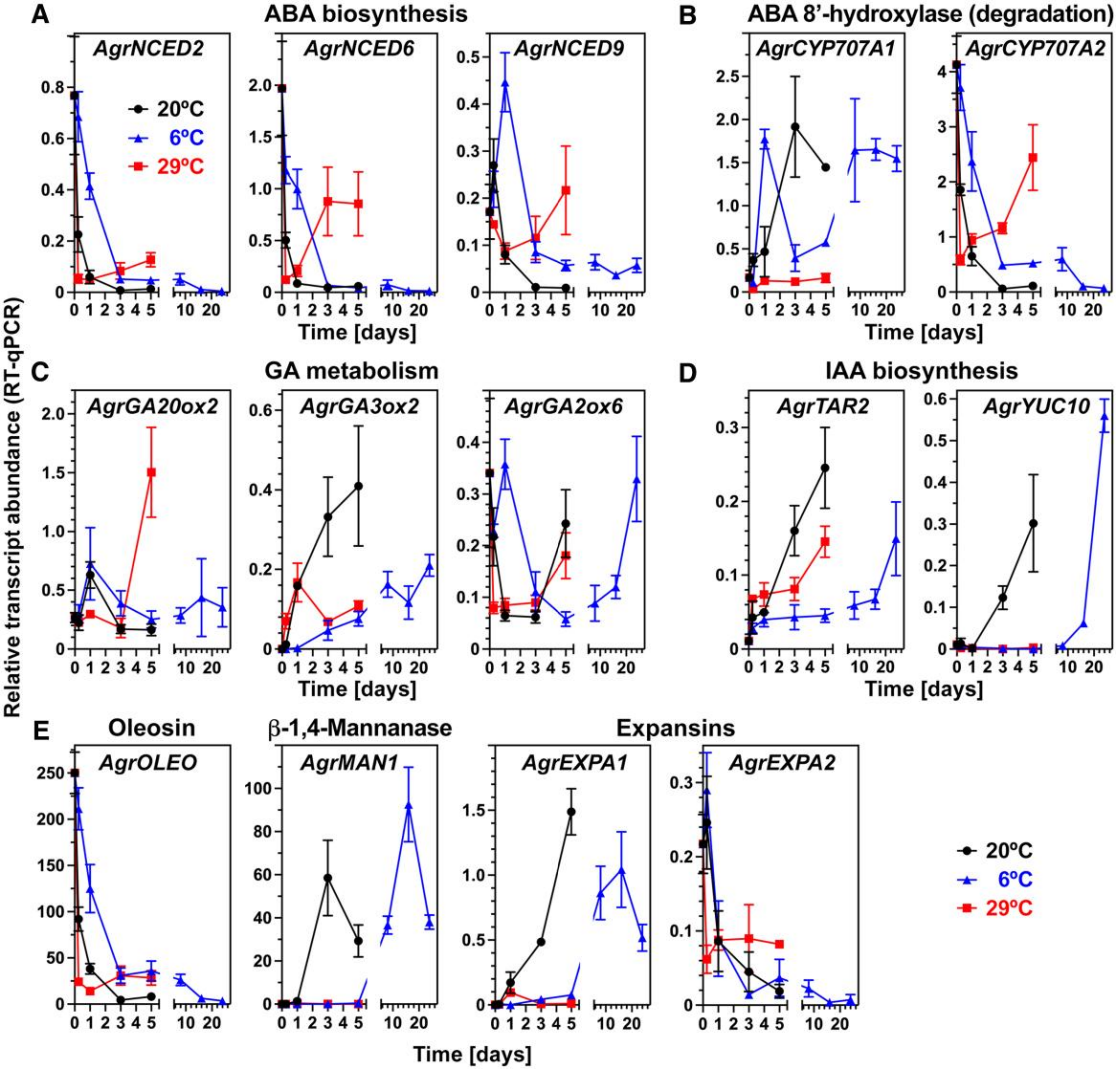
The effects of suboptimal (6 °C), optimal (20 °C), and supraoptimal (29 °C) temperatures on the expression of key genes in celery cultivar Victoria fruits imbibed in continuous white light. (A) Whole-fruit relative transcript abundance patterns (RT–qPCR) for abscisic acid (ABA) biosynthesis genes encoding 9-*cis*-epoxycarotenoid dioxygenases (NCED). (B) RT–qPCR for ABA catabolism genes encoding ABA 8′-hydroxylases (CYP707A). (C) RT–qPCR for gibberellin (GA) metabolism genes encoding GA 20-oxidase (GA20ox), GA 3-oxidase (GA3ox), and GA 2-oxidase (GA2ox). (D) RT–qPCR for indole-3-acetic acid (IAA) biosynthesis genes encoding tryptophan aminotransferase TAR2 and YUCCA flavin monooxygenases (YUC10). (E) RT–qPCR for oleosin, β-1,4-mannanase, and α-expansin genes. Mean values ±SEM of three biological replicate RNA samples.

To evaluate whether expression of key genes in celery germination (Walker *et al.*, 2021) was affected by imbibition temperature, we performed transcript expression analyses by RT–qPCR at 6 °C (chilling), 20 °C (optimal), and 29 °C (thermoinhibition) as representative temperatures (Fig. 6). For this we sampled at all three temperatures at the same physical time points: 0.25, 1, 3, and 5 d. In addition, at 6 °C, we sampled at 8, 16, and 24 d, which were determined by our quantitative analysis of embryo growth (Fig. 4A). These physiological time points were defined by the quantitative analysis of embryo growth to represent the same growth stages as at 1, 3, and 5 d at 20 °C, respectively. In the established thermal time models for embryo growth and germination (Fig. 4B), these physiological times also represent similar heat units: ∼20 °C·d (8 d at 6 °C and 1 d at 20 °C), ∼50 °C·d (16 d at 6 °C and 3 d at 20 °C), and ∼80 °C·d (24 d at 6 °C and 5 d at 20 °C). The embryo growth stages (Walker *et al.*, 2021) associated with these physiological times for 20 °C versus 6 °C are as follows: (I) 1 d and 8 d are before the initiation of embryo growth; (II) 3 d and 16 d are midway through growth; and (III) 5 d and 24 d are towards the completion of the growth phase (Fig. 4A).

Walker *et al*. (2021) demonstrated that at 20 °C ABA levels declined rapidly upon imbibition of celery fruits and, consistent with this, the transcript abundances for the key ABA biosynthesis 9-*cis*-epoxycarotenoid dioxygenase (NCED) genes declined and transcripts of *AgrCYP707A1* encoding ABA 8′-hydroxylase involved in ABA degradation accumulated, with transcripts being more abundant in the endosperm as compared with the embryo. In contrast to 20 °C and consistent with a role for ABA biosynthesis in thermoinhibition, transcripts of *AgrNCED2*, *AgrNCED6*, and *AgrNCED9* accumulated and the *AgrCYP707A1* transcript abundances remained low at 29 °C (Fig. 6A, B). NCED expression also declined and *AgrCYP707A1* transcripts accumulated in celery fruits imbibed at 6 °C. *AgrCYP707A2* transcripts exhibited a contrasting pattern, with decline at 6 °C and 20 °C and accumulation at 29 °C (Fig. 6B). It was proposed earlier (Walker *et al.*, 2021) that this CYP707A gene is involved in feedback regulation to control ABA degradation. It was also demonstrated that both *AgrCYP707A1* and *AgrCYP707A2* are up-regulated by light and down-regulated by darkness (Li *et al.*, 2022). Taken together, the NCED and CYP707A trancript abundance patterns (Fig. 6A, B) suggest that ABA degradation occurs over the entire suboptimal temperature range and that thermoinibition is associated with ABA accumulation.

Increasing bioactive GA levels were associated with the transient expression of the GA 20-oxidase and GA 3-oxidase biosynthesis genes *AgrGA20ox2* and *AgrGA3ox2* in the embryo of celery fruits imbibed at 20 °C (Walker *et al.*, 2021). The *AgrGA20ox2* transcript levels were low in dry fruits, transiently peaked after 1 d of imbibition at both 6 °C and 20 °C, before declining to lower levels by 3 d (Fig. 6C). At 29 °C, this 1 d transient peak was suppressed and instead *AgrGA20ox2* was up-regulated at 5 d. At 20 °C, *AgrGA3ox2* was expressed at a low level in dry fruits and accumulated ∼36-fold by 5 d (Fig. 6C). At 6 °C, *AgrGA3ox2* transcripts accumulated far more slowly, reaching their highest observed level by 24 d. At 29 °C, *AgrGA3ox2* was initially induced in a similar manner to at 20 °C, but by 3 d its increase was suppressed (Fig. 6C). GA 2-oxidases are key enzymes of GA catabolism and, in celery fruits imbibed at 20 °C, *AgrGA2ox6* is more highly expressed in the endosperm compared with the embryo (Walker *et al.*, 2021). *AgrGA2ox6* was highly expressed in dry fruits and, upon imbibition at both 20 °C and 29 °C, fell to low levels by 1 d, and remained low at 3 d before expression increased (Fig. 6C). At 6 °C, expression initially declined in a similar manner to the higher temperatures, before it was transiently up-regulated at day 1; subsequently expression remained low before rising to a second peak at 24 d. Taken together, the *AgrGA3ox2* trancript abundance patterns (Fig. 6C) suggest that the bioactive GA production and accumulation in celery fruits imbibed at 20 °C (Walker *et al.*, 2021) also occurs in celery fruits imbibed at 6 °C, but not at 29 °C.

Walker *et al*. (2021) demonstrated that at 20 °C the indole-3-acetic acid (IAA) contents in celery fruits rapidly declined upon imbibition (day 1) and subsequently increased during embryo growth and radicle emergence. Most of this IAA was localized in the endosperm where its levels remained roughly constant over the study period. In contrast to this, the IAA contents within the embryo increased ∼8-fold in association with its growth. Key IAA biosynthesis genes, such as TAR2 tryptophan aminotransferase and YUCCA flavin monooxygenases, exhibited embryo-associated expression patterns well correlated with the observed IAA contents in celery fruits imbibed at 20 °C. It is shown in Fig. 6D that expression of *AgrTAR2* was lowest in dry fruits and increased upon imbibition at 20 °C, reaching its highest level after 5 d. At 29 °C, *AgrTAR2* expression followed a similar pattern, though the increase was slower. At 6 °C, *AgrTAR2* remained expressed at a lower level up to 16 d before accumulating. Similarly, *AgrYUC10* was expressed at a low level in dry fruits, and upon imbibition at 20 °C expression increased >70-fold by 5 d. At 6 °C, this increase was delayed, with transcripts negligible up to 8 d before accumulating (Fig. 6D). At 29 °C, the *AgrYUC10* transcript abundances remained very low throughout the observation period. Taken together, the *AgrTAR2* and *AgrYUC10* trancript abundance patterns (Fig. 6D) suggest that IAA production and accumulation in celery fruits imbibed at 20 °C (Walker *et al.*, 2021) also occur in celery fruits imbibed at 6 °C, but not at 29 °C.

Oleosins are oil body-associated proteins that are involved in the developmental-related synthesis of seed lipid bodies, a major form of seed stored resources (Huang, 1996). Consistent with storage protein and oil body mobilization and GA-induced endosperm degradation, the *AgrOLEO* transcript abundance was high in dry celery fruit endosperm and fell rapidly at 20 °C upon imbibition (Fig. 6E; Walker *et al.*, 2021). This expression pattern was also evident at 29 °C and at 6 °C, with the high temperature accelerating and the low temperature decelerating the decline, respectively (Fig. 6E). Endo-β-1,4-mannanases (MANs) are enzymes involved in the hydrolysis of endosperm cell wall β-1,4-mannans; their expression and activities have been shown to be associated with endosperm degradation required for the embryo growth in MD carrot seeds (Homrichhausen *et al.*, 2003). Expression of celery *AgrMAN1* was low in the dry fruits and, after, 1 d of imbibition at 20 °C, accumulated ∼1500-fold, with a peak at day 3 (Fig. 6E; Walker *et al.*, 2021). In contrast to this, at 29 °C, *AgrMAN1* expression was suppressed. During celery fruit imbibition at 6 °C, *AgrMAN1* transcript abundances remained low until day 5, and subsequently increased ∼2500-fold, with a peak at day 16 (Fig. 6E). Expansins are proteins that facilitate cell wall extension, weakening, and disassembly. Expansin genes are up-regulated by GA and auxin during embryo and seedling cell growth and division, as well as during endosperm and fruit tissue weakening (Chen *et al.*, 2001; Yan *et al.*, 2014; Steinbrecher and Leubner-Metzger, 2017). We identified two endosperm-expressed celery α-expansins which showed contrasting transcript accumulation patterns in imbibed fruits at 20 °C (Walker *et al.*, 2021). *AgrEXPA2* was expressed at a low level in dry fruits and declined further at all temperatures (Fig. 6E). *AgrEXPA1* was expressed at a low level in dry fruits and accumulated ∼5000-fold by 5 dduring imbibition at 20 °C , whilst at 29 °C its expression was suppressed. At 6 °C, *AgrEXPA1* transcript abundances remained low up to 5 d, and subsequently increased ∼3000-fold, with a peak at 16 d (Fig. 6E). Taken together, the temperature-regulated expression of *AgrEXPA1* and *AgrMAN1* in the endosperm was associated in a thermal time-compliant manner at suboptimal (6 °C; ∼50 °C·d at 16 d) and optimal (20 °C; ∼50 °C·d at 3 d) temperatures, and was surpressed at 29 °C, a temperature which also inhibits embryo growth and germination.

## Discussion

### High temperatures and darkness interact with hormonal pathways to inhibit germination through suppression of embryo growth

Thermoinhibition is the suppression of seed germination at high imbibition temperatures, but thermoinhibited seeds will germinate when transferred to optimal temperatures (Argyris *et al.*, 2008; Michael *et al.*, 2023; Piskurewicz *et al.*, 2023). The mechanisms underpinning the delayed or severely inhibited seed germination by thermoinhibition were studied in species which produce seeds with fully developed embryos such as *A. thaliana* (at 34 °C) and lettuce (at 35–38 °C). These works identified that thermoinhibition is mediated by complex alterations of hormone metabolism and signalling (Dutta *et al.*, 1997; Nascimento *et al.*, 2001; Argyris *et al.*, 2008; Toh *et al.*, 2008; Huo *et al.*, 2013), that the endosperm is a temperature-sensing tissue that implements thermoinhibition through phytochrome B (phyB) and PIF-mediated ABA accumulation (Piskurewicz *et al.*, 2023), and that thermoinhibition inhibits embryo growth and endosperm weakening (Nascimento *et al.*, 2001; Steinbrecher and Leubner-Metzger, 2017). The phenomenon of thermoinhibition is known for species with MD fruits including for the germination of carrot (at 35 °C, Nascimento *et al.*, 2013) and celery (at 32 °C, Biddington and Thomas, 1978), but the effects on embryo growth and the underpinning hormonal and molecular mechanisms were not studied. We demonstrated here that thermoinhibition causes a complete block to germination at 29 °C which is achieved through the complete suppression of embryo growth (Figs 1–4). Treatment of celery fruits imbibed at 29 °C with GA or the carotenoid/ABA biosynthesis inhibitor FLU reverted the thermoinhibition of embryo growth and germination. Treatment with FLU, but not appreciably GA, increased T_c_ for germination by ∼8 °C from ∼28 °C to ∼35 °C (Fig. 3), and treatment with FLU plus GA fully reverted germination G_max_ and speed at 29 °C to optimal temperature (20 °C) responses (Fig. 5). While 100 µM ABA inhibits germination in the optimal and supraoptimal temperature range, treatment with 100 µM ABA does not affect embryo growth at 20 °C in celery (Fig. 5C; Walker *et al.*, 2021) and carrot (Homrichhausen *et al.*, 2003) fruits.

In celery fruits imbibed at optimal temperature (20 °C), ABA degradation (mainly in the endosperm) and GA biosynthesis (mainly in the embryo) (Walker *et al.*, 2021) are associated with the decline in transcript abundances for ABA biosynthesis genes *AgrNCED2*, *AgrNCED6*, and *AgrNCED9*, encoding NCEDs, and the up-regulation of the ABA degradation gene *AgrCYP707A1*, encoding ABA 8′-hydroxylase (Fig. 6). In contrast to this, during thermoinhibition (29 °C) the three celery *NCED* genes are up-regulated and the induction of *AgrCYP707A1* is inhibited, suggesting that ABA levels are increasing during thermoinhibition, and are fine-tuned in the thermoinhibited state by *AgrCYP707A2* (Fig. 6). As these ABA-related genes are mainly expressed in the endosperm (Walker *et al.*, 2021), we speculate that thermoinhibition effects on embryo growth and germination are, at least in part, manifested via ABA production in the endosperm. Celery embryo growth inhibition during thermoinhibition (29 °C) is, however, not simply caused by ABA accumulation which directly inhibits embryo growth, because treatment with 100 µM ABA at optimal temperature (20 °C) does not affect embryo growth (Fig. 5C; Walker *et al.*, 2021). There is also evidence that an unknown germination inhibitor distinct from ABA accumulates in thermoinhibited celery fruits (Thomas *et al.*, 1986). Treatment with ABA also shifted the temperature germination optimum for celery fruits from 21–22 °C to 18 °C, suggesting changes in ABA sensitivity (Fig. 3). In agreement with this, increasing temperatures also increase the sensitivity to ABA, and ABA signalling is invoved in lettuce thermoinhibition (Roth-Bejerano *et al.*, 1999; Eckhardt *et al.*, 2024). A limitation of bioactive GAs is also probable, since exogenous GA promotes embryo growth at high temperatures, and thermoinhibition is associated with suppression of the GA biosynthesis gene *AgrGA3ox*, encoding a GA3ox which converts inactive GA precursors into bioactive GAs (Fig. 6; Walker *et al.*, 2021). Moreover, auxin biosynthesis genes (*AgrTAR2* and *AgrYUC10*), as well as auxin transport, play positive roles in regulating embryo growth in celery fruits (Walker *et al.*, 2021). These auxin biosynthesis genes are inhibited during thermoinhibition, suggesting that auxin biosynthesis is probably also suppressed.

A role for GA limitation in thermoinhibited celery fruits is further supported by the fact that known GA-responsive transcripts, as well as those involved in the GA-induced process for endosperm cell wall weakening and degradation required for embryo growth, MAN (*AgrMAN1*) and expansin (*AgrEXPA1*), were suppressed at high temperature (Fig. 6). GA treatment of celery fruits at optimal temperature (20 °C) promoted earlier and/or enhanced expression of *AgrMAN1* and *AgrEXPA1* in the endosperm of celery fruits very early during imbibition (Walker *et al.*, 2021). Treatment of MD celery fruits imbibed at 20 °C with 100 µM ABA did not appreciably affect *AgrMAN1* and *AgrEXPA1* expression and embryo growth (Walker *et al.*, 2021). MAN transcript expression (*DcMAN1*) in the micropylar endosperm half and the increase in enzyme activity in MD carrot fruits imbibed at optimal temperature were, like embryo growth, also not inhibited by 100 µM ABA (Homrichhausen *et al.*, 2003). In contrast to this, the increase in MAN enzyme activity in lettuce fruits imbibed at optimal temperature was inhibited by ABA, and was also inhibited by thermoinhibition (32 °C), but GA treatment reversed this inhibition (Dutta *et al.*, 1997). Inhibition of MAN activity accumulation at high temperature, thereby inhibiting endosperm degradation required for embryo growth, could therefore be a key mechanism by which thermoinhibition inhibits the germination of celery and carrot fruits in a GA-induced and ABA-insensitive manner. Interestingly, a seasonal pattern of MAN activity detected in *Jeffersonia dubia* MPD fruits was related to temperature changes and associated with the release of physiological dormancy, embryo growth, endosperm degradation, and germination (Kwon *et al.*, 2025).

In contrast to the carotenoid/ABA biosynthesis inhibitor FLU, GA treatment alone only partly reversed the thermoinhibition of embryo growth, germination speed, and G_max_ (Figs 2–5). Similarly, GA treatment could only partly replace the light signal to trigger germination of celery fruits imbibed in darkness at optimal temperature (Fig. 1; Walker *et al.*, 2021). Embryo growth analysis of dark-imbibed celery fruits demonstrated that although the GA treatment induced embryo growth, this growth did not reach the critical E:F ratio required for the completion of germination (Fig. 1D). Phytochrome B integrates light and temperature signals in *A. thaliana* (Legris *et al.*, 2016; Piskurewicz *et al.*, 2023), and is known to be involved in germination of light-requiring species such as lettuce (Dutta *et al.*, 1997; Roth-Bejerano *et al.*, 1999; Argyris *et al.*, 2008). The germination of many Apiaceae species is light requiring and, as for celery (Fig. 1D), the embryo growth of *Sison amomum* (MD), *Angelica sylvestris* (MPD), *Anthriscus sylvestris* (MPD), and *Chaerophyllum temulum* (MPD) required light and did not occur in darkness (Baskin *et al.*, 2000; Vandelook *et al.*, 2007; Van Assehe *et al.*, 2011). In contrast to this, embryo growth and germination of *Conopodium majus* (MD) occurred in light and darkness, but only at 0–5 °C, and was inhibited at 10 °C (Blandino *et al.*, 2019). *Apium graveolens* (celery) fruits may have retained a low level of residual physiological dormancy which does not affect G_max_, but reduces the germination speed (Fig. 1). Similarly, freshly harvested *Cyclospermum* (*Apium*) *leptophyllum* (wild close relative of celery) and *Pastinaca sativa* (parsnip) MD fruit batches may have a low level of residual physiological dormancy (thus, MPD), but the proportion of these MPD fruits varies between batches and populations (Baskin and Baskin, 1979; Walck *et al.*, 2008) and, in contrast to celery (Fig. 1), G_max_ was also affected in these cases. These examples suggest that the distinction between MD and MPD is probably more fluid and affected by environmental cues during seed/fruit development.

### Suboptimal temperatures cause a delay in the physiological transition that is associated with the initiation of embryo growth

In contrast to what is observed at higher temperatures, suboptimal temperatures do not suppress celery embryo growth and germination, rather embryo growth is delayed over the entire suboptimal temperature range between 6 °C and 20 °C (Figs 3, 4). This is mainly achieved by delaying the initiation of embryo growth, leading to delays in the onset of the completion of germination. This reduced the germination speed, but without appreciably affecting G_max_ and the shape of the germination curves. Thermal time modelling of embryo growth and germination (Fig. 4B) revealed that they have the same base temperature (∼3 °C) and embryo growth precedes germination with an ∼1.4-fold lower Θ_cold50%_ for the median percentile (61.2 °C·d versus 86.8 °C·d, Fig. 4D). In contrast to celery where the initiation of embryo growth was delayed and the speed of embryo growth to reach the critical E:F ratio was reduced at 6 °C compared with higher temperatures, in the MD fruits of *C. majus* embryo growth was promoted by 0–5 °C and blocked by 10 °C (Blandino *et al.*, 2019, 2025). Also in contrast to celery, in the MD fruits of *S. amomum*, embryo growth was highest at 5 °C, slower at 10 °C, and blocked at 23 °C (Van Assehe *et al.*, 2011). In contrast to the situation at optimal and supraoptimal temperatures, treatment of celery fruits with GA, ABA, or FLU did not appreciably affect celery germination (T_b_, Θ_cold50%_, and T_o_) in the suboptimal temperature range (Figs 3, 4). The notable delay in the initiation of embryo growth suggests that lower temperatures may cause a delay in the hormonally controlled physiological transition that is required for embryo growth initiation. To test this hypothesis, the expression of key genes previously identified to be associated with this transition at optimal (20 °C) temperature (Walker *et al.*, 2021) was compared with their expression at suboptimal (6 °C) temperature (Fig. 6). In contrast to thermoinhibition which blocks celery embryo growth, cell wall-remodelling gene expression, and germination, chilling temperatures (6–20 °C) delay the initiation of embryo growth and thereby reduce the expression of key cell wall-remodelling genes and germination speed in a linear manner according to thermal time.

Cold stratification (i.e. imbibition at low temperature) of seeds with a fully developed embryo release dormancy and promotes germination through modulating GA and ABA metabolism and sensitivity (Finch-Savage and Leubner-Metzger, 2006). In *A. thaliana* seeds, cold stratification at 4 °C has been shown to enhance the expression of the GA20ox and GA3ox GA biosynthetic genes as well as GA-responsive genes, which leads to the early accumulation of bioactive GAs in response to the cold stratification (Ogawa *et al.*, 2003; Yamauchi *et al.*, 2004). In contrast to this, we did not observe any enhancement of the quantified transcript abundances for the GA biosynthesis genes (*AgrGA20ox2* and *AgrGA3ox2*) in celery fruits imbibed at 6 °C compared with 20 °C (Fig. 4C). Instead, *AgrGA3ox2* expression was delayed considerably and remained at a lower level. Also, transcripts of the GA2ox catabolism gene *AgrGA2ox6* were transiently up-regulated early on at 6 °C, which did not occur at 20 °C and 29 °C. This strongly suggests that accumulation of bioactive GAs in celery fruits in response to low temperature is unlikely and, that in contrast to *A. thaliana*, GA inactivation is promoted in celery fruits in response to the low temperature. However, GA limitation at suboptimal temperatures is probably not the only factor for the delayed initiation of embryo growth, since exogenous GA did not promote germination at any suboptimal temperatures (Fig. 3). This suggests that at lower temperatures there is likely to be reduced sensitivity to GA in celery fruits as well, which is in contrast to *A. thaliana* seeds where chilling increases GA sensitivity (Ogawa *et al.*, 2003; Yamauchi *et al.*, 2004).

Expansins and cell wall-remodelling enzymes such as MAN are involved in endosperm weakening in seeds with fully developed embryos, and their genes are often GA responsive (Chen and Bradford, 2000; Nonogaki *et al.*, 2000; Ogawa *et al.*, 2003; Yan *et al.*, 2014; Steinbrecher and Leubner-Metzger, 2017). In MD fruits, an increase in MAN enzyme activity in the endosperm of carrot, and expression ABA-insensitve expression of the *DcMAN1* and *AgrMAN1* genes in the endosperm of carrot and celery were associated with embryo growth (Homrichhausen *et al.*, 2003; Walker *et al.*, 2021). GA treatment of celery fruits at optimal temperature (20 °C) promoted earlier and/or enhanced expression of *AgrMAN1* and *AgrEXPA1* in the endosperm of celery fruits very early during imbibition (Walker *et al.*, 2021). Consistent with a later initiation of embryo growth and delayed germination at suboptimal temperatures, the induction of *AgrMAN1* and *AgrEXPA1* expression was significantly delayed (Fig. 6E). The *AgrMAN1* and *AgrEXPA1* transcript abundances peaked much later in a thermal time-compliant manner at suboptimal (6 °C; ∼50 °C·d at 16 d) and optimal (20 °C; ∼50 °C·d at 3 d) temperatures.

Cold stratification has also been shown to modify ABA metabolism and signalling, and is associated with a decline in *A. thaliana* seed ABA contents (Kushiro *et al.*, 2004; Yan *et al.*, 2014). The transcript abundances of the NCED ABA biosynthesis genes declined in a very similar manner in imbibed celery fruits at 6 °C and 20 °C (Fig. 6A). The expression of ABA 8′-hydroxylase *AgrCYP707A1* is up-regulated considerably upon imbibition at 20 °C, and this is associated with a decline in endogenous ABA levels in association with the initiation and progression of embryo growth (Walker *et al.*, 2021). At 6 °C, *AgrCYP707A1* transcript accumulation exhibited an early and a late peak, suggesting that delayed induction and progression of embryo growth was associated with ABA degradation (Fig. 6B). These observations suggest that enhanced ABA biosynthesis does not play a role in the delay of embryo growth at low temperatures, which is consistent with the finding that 100 µM ABA does not affect embryo growth at optimal temperature. We show here that at 6 °C there was a delay in the expression of auxin biosynthesis genes (*AgrTAR2* and *AgrYUC10*), their later increases again occurring in association with the initiation and progression of embryo growth at this suboptimal temperature (Fig. 6D). Auxins play cardinal roles in the promotion of cell expansion and division, including in celery fruits where increasing IAA contents are associated with embryo growth driven by cell expansion and division (Walker *et al.*, 2021). The delayed expression of auxin biosynthesis genes therefore supports the hypothesis that embryo cell expansion and division are slowed down by suboptimal temperatures.

### Embryo growth in imbibed celery fruits is temperature dependent and regulates germination timing and capacity

Population-based thermal time threshold modelling of MD celery fruits indicated that the cardinal temperatures for embryo growth and germination were roughly identical (Fig. 4). The suboptimal temperature range between the base (T_b_ ∼3 °C) and optimal (T_o_ 21–22 °C) temperatures was characterized by a roughly linear increase with increasing temperature for embryo growth (initiation and speed) and germination (onset of radicle protrusion and speed) rates. This intimate connection between both traits suggests that germination timing depends on the temperature-dependent speed of embryo growth to reach the critical EF ratio required for the completion of germination by radicle emergence. Chilling therefore seems to simply decelerate the identified mechanisms by which celery embryo growth and endosperm degradation are achieved (Walker *et al.*, 2021). Conversely, as we move from optimal through supraoptimal temperatures, thermoinhibition causes a complete block to germination at 29 °C (Figs 3, 4). Analysis of embryo growth across supraoptimal temperatures highlights that celery thermoinhibition is achieved through the complete suppression of embryo growth. The initiation of embryo growth is therefore regulated by mechanisms that involve the integration of temperature signals and functions to regulate germination timing (chilling, control of embryo growth speed) and capacity (thermoinhibition, block of embryo growth).

The initial and the critical relative embryo sizes (E:F, ‘embryo to fruit’ size ratios) of the three MD celery cultivars were ∼0.32 and ∼0.81, respectively (Figs 1, 2, 4). As a comparison, the initial and final embryo to seed length ratios of 43 wild Apiaceae species from temperate climate habitats were ∼0.19 (range 0.04–0.90) and ∼0.79, respectively (Visscher *et al.*, 2022 ). These wild species differed from the Apiaceae crops celery (this work) and carrot (Corbineau *et al.*, 1995; Finch-Savage *et al.*, 1998 ) in that most of them had MPD and that their thermal profile was significantly shifted towards colder temperatures. Among the few wild Apiaceae species with MD is *C. majus* for which embryo growth from an initial 0.12 to a final ∼1 E:S ratio was promoted by low temperatures (0–5 °C) and arrested above 10 °C (Blandino *et al.*, 2019). Analysis of the embryo growth of nine *C. majus* populations revealed intraspecific variation in thermal thresholds for the base (−6.6 °C to −2.7 °C), optimal (2.5 °C to 5.5 °C), and ceiling (12.0 °C to 20.5 °C) temperatures (Blandino *et al.*, 2025). Another example for MD is *S. amomum* for which embryo growth (from initial 0.35 to critical 0.82 E:S ratio) and germination occurred at low temperatures (5–10 °C) (Van Assehe *et al.*, 2011). Apiaceae crops including celery and carrot seem to have higher thermal thresholds for germination compared with wild Apiaceae species.

In summary, celery fruits therefore provide an excellent system to study the thermal and hormonal mechanisms of MD in the absence of the physiological dormancy component. From works at optimal germination temperature (Walker *et al.*, 2021), it is clear that a complex interaction between GA, auxin, and ABA metabolism and changes in the tissue-specific sensitivities to these hormones controls the unique germination programme of MD celery fruits. The embryo growth inside the celery fruit is not simply the completion of embryogenesis or *A. thaliana*-equivalent post-embryogenesis growth, but a distinct process as revealed by the hormonal mechanisms, embryo–endosperm interactions, and the spatiotemporal expression patterns of corresponding genes. A clear role for crosstalk between the embryo and the endosperm is apparent, with GA released by the embryo inducing responsive genes in the endosperm. Moreover, a biphasic nature of endosperm breakdown became apparent (this work; Walker *et al.*, 2021), with the main bulk of endosperm breakdown being less ABA sensitive and associated with resource mobilization to facilitate cavity formation and to fuel embryo growth (phase II). In contrast to this, the late stage (phase III) is strongly ABA sensitive to control radicle protrusion probably by restraint weakening of the micropylar endosperm and pericarp as is known from ND/PD seeds (Yan *et al.*, 2014; Chahtane *et al.*, 2017; Steinbrecher and Leubner-Metzger, 2017).

Our work with celery fruit responses to suboptimal and supraoptimal temperatures (Figs 1, 6) revealed distinct hormone–temperature interactions and distinct underpinning mechanisms for reducing embryo growth. During thermoinhibition, the initiation of embryo growth is suppressed in a GA–ABA-regulated manner, and the block to germination is therefore achieved by preventing the embryo inside the imbibed fruit from growing to the critical size. Darkness also acts by supressing embryo growth. During thermoinhibition, the expression of *AgrMAN1*, *AgrEXPA1*, and *AgrYUC10* is blocked, the expression of *AgrGA3ox2* and *AgrCYP707A1* is reduced, and the expression of ABA biosynthesis genes (NCEDs) is enhanced. In contrast to this, during chilling, the initiation of embryo growth is delayed in a thermal time-compliant manner. This involves thermal time-compliant delays in the expression of key genes in endosperm weakening/degradation (*AgrMAN1* and *AgrEXPA1*) to generate the gap required for embryo growth, and auxin and GA biosynthesis important for cell expansion and division, necessary for embryo growth and a reduction in GA and ABA sensitivity of the growing embryo.

## Data Availability

All data presented or analysed in this published article are available online through figshare https://doi.org/10.17637/rh.28741217.v1.
